# Women’s lived experiences of learning to live with osteoporosis: a longitudinal qualitative study

**DOI:** 10.1186/s12905-017-0377-z

**Published:** 2017-03-09

**Authors:** Carrinna A. Hansen, Bo Abrahamsen, Hanne Konradsen, Birthe D. Pedersen

**Affiliations:** 1 Institute of Clinical Research, Faculty of Health Sciences, University of Southern Denmark, Campusvej 55, 5230 Odense M, Denmark; 2 Department of Medicine C, Gentofte University Hospital, Kildegårdsvej 28, 2900 Hellerup, Denmark; 3 Department of Medicine, Holbæk Hospital, 4300 Holbæk, Denmark; 4 Research Unit, Gentofte University Hospital, Kildegårdsvej 28, 2900 Hellerup, Denmark

**Keywords:** Chronic disease, Denmark, Lived experiences, Longitudinal, Medical treatment, Newly-diagnosed, Osteoporosis, Phenomenological-hermeneutic, Qualitative research, Women

## Abstract

**Background:**

A vast amount of literature exists concerning pharmaceutical adherence in osteoporosis. However, the process of learning to live with osteoporosis over time remains largely unknown. The purpose of this study was to gain a deeper understanding of the continued process of how women learn to live with osteoporosis. Our objective was to explore what characterizes women’s experiences of living with osteoporosis during the first year after diagnosis, when patients are prescribed anti-osteoporotic treatment, without having experienced an osteoporotic fracture.

**Methods:**

Forty-two narrative qualitative interviews were conducted with fifteen recently diagnosed Danish women. A longitudinal design was chosen since this allows an investigation of the perspective over time. The interviews were conducted in the period of March 2011 to August 2012. Data were analyzed using a phenomenological-hermeneutic interpretation of text. No medical records were available for the researchers. All information with the exception of T-score was self-reported.

**Results:**

The participants’ experiences could be described in two key themes developed through the analysis: 1) “To become influenced by the medical treatment” which consisted of two sub-themes “taking the medication”, and “discontinuing the medication”. 2) “Daily life with osteoporosis”, which was characterized by three sub-themes: “interpretation of symptoms”, “interpretation of the scan results” and “lifestyle reflections”. The results highlighted that learning to live with osteoporosis is a multifaceted process that is highly influenced by the medical treatment. In some cases, this is a prolonged process that can take around one year.

**Conclusions:**

The results suggest a need for improved support for individual women during the complex process of learning to live with osteoporosis. The study adds new knowledge that can be useful for healthcare professionals taking a health-oriented stance when supporting women in self-management of their illness. Further investigations of lived experiences over time in the field of osteoporosis research are therefore needed.

**Electronic supplementary material:**

The online version of this article (doi:10.1186/s12905-017-0377-z) contains supplementary material, which is available to authorized users.

## Background

Osteoporosis is an increasing major public health problem [1–3] which affects hundreds of millions of people worldwide [4]. This condition is, however, greatly underdiagnosed and undertreated [3–5], leading to an increased need for early detection, treatment and osteoporosis education to prevent deterioration and disability [4, 5], as well as the need to improve the prognosis [6], the quality of life [7–9] and to prevent premature death [3, 10, 11]. Osteoporosis is a chronic condition that is generally treatable in otherwise healthy individuals but it can also become severely debilitating if left untreated [2, 12].

### Osteoporosis definition and causal explanations

The current definition of osteoporosis for post-menopausal women was first proposed in 1944 by a Task Force under the World Health Organization (WHO) [13]:

“Severe osteoporosis (established osteoporosis). A bone mineral density (BMD) that is more than 2.5 standard deviation (SD) below the young adult mean in the presence of one or more fragility fractures” pp. 6 [14].

The diagnosis is made on the basis of a Dual-energy X-ray absorptiometry (DXA) scan and a quantitative assessment of the BMD, which is a determinant of bone strength. BMD is measured as a T-score in lumbar spine or hip. Low BMD can be a risk factor but does not always lead to fractures. The risk of fracture is highly influenced by other factors such as age, heredity, Body Mass Index, weight-bearing exercise and lifestyle [3, 15]. The most common physical consequences of osteoporosis are compression fractures of the spine, fracture at the hip, distal forearm and proximal humerus.

### Epidemiology and fracture

The prevalence of female osteoporosis in the five largest countries in Europe (France, Germany, Italy, Spain and the UK) is high, with 21% of women aged 50–84 years (representing more than 12 million) being affected in these countries [3]. In Denmark, there are no accurate figures of how many individuals are affected by osteoporosis, partly because the disease is often asymptomatic in the early stages and also because guidelines do not consider systematic screening initiatives to be justified [2, 16, 17]. However, Vestergaard et al. estimated in 1995 that 40.8% of women and 17.7% of men over 50 years of age have osteoporosis in Denmark [5]. As a result of the increase in the size of the aging population [15, 18] along with an increased awareness of the condition, the number of men and women diagnosed as living with osteoporosis has also increased in recent decades.

### Osteoporosis, consequences and challenges - a continued public health problem

The consequences of osteoporosis include physical, psychological, economic and societal factors [2, 10, 19]. It is a continued challenge to promote bone health and to prevent osteoporotic fractures [6, 7, 20] in a growing worldwide elderly population. This challenge is exacerbated by the fact that many individuals do not follow the prescribed anti-osteoporotic treatment as recommended [21–26]. Despite extensive investigations of this phenomenon, it seems that the underlying causal explanations of medication failure (such as quitting treatment early or low compliance to treatment leading to lack of treatment efficacy) [23] have not yet been fully explored. It has been suggested that the research in the field of fracture prevention treatment needs to focus on patients’ own perceptions [23, 27, 28]. This type of research calls for the use of qualitative research methods to make the voices of those involved heard.

### A thorough understanding of women’s experience of osteoporosis

Studies have found that being diagnosed with osteoporosis may lead to psychological and physical consequences for the individuals, impacting their quality of life [8, 29–35]. In addition to this, patients may find it difficult to make sense of the diagnosis and its implications for their current and future health [8]. These difficulties may be associated with the emotional challenges of handling the knowledge of the fracture risk [29], as well as thoughts about osteoporosis and risk perception influenced by stereotypes of bodily deterioration and founded on worst-case scenarios [30] and interpretations of information derived from the DXA scan as body fragility and risk [31]. There are a variety of ways in which women with osteoporosis perceive themselves and manage their chronic illness and ageing, arguing that structural and psychological determinants of health behavior need to be understood in order to better understand and manage the disease [36, 37].

In the current study, we chose a qualitative method in order to supplement former studies of osteoporosis. This was chosen in order to explore the patient’s perspective of osteoporosis, and to detect areas where improvements in treatment could be made. The objective was to explore what characterizes women’s experiences of living with osteoporosis in the first year after diagnosis, when patients are prescribed anti-osteoporotic treatment, without experiencing an osteoporotic fracture.

## Methods

This qualitative study used a phenomenological-hermeneutic approach of narratives and interpretation [38–40]. The French philosopher Paul Ricoeur’s work is commonly regarded as a bridge between the philosophies of phenomenology and hermeneutic [41]. In this interpretation, the structures of subjective experience and consciousness, and the self and its realization are interpreted through the hermeneutic circle [42]. The approach was chosen in order to be open towards patients’ perspectives when exploring women’s experiences, beliefs, attitudes, behavior and lifestyle when living with a new diagnosis of osteoporosis. Additionally, a longitudinal design was chosen because it allows the tracking of changes over time, and for examining development of patterns and changes. This may provide a more comprehensive level of information regarding individual experiences and perceptions [43].

The initial sampling of women 65 years or older who attended DXA scan took place at two hospitals in two different regions of Denmark. The methods of recruitment and discussion of non-participators have been reported elsewhere [44, 45]. In the present study, we investigated the experiences during the conversion to new life circumstances with osteoporosis.

### Participants

Women were contacted when they attended a DXA scan at one of the two participating hospitals during the period of January to April 2011. Fifteen participants were included consecutively according to inclusion and exclusion criteria, the inclusion criteria being aged 65 years or older, with a new diagnosis of osteoporosis and a DXA scan showing a T-score below −2.5 (lower back or hip) [13], as well as no previous known osteoporotic fracture and self-reported confirmation of at least one of the known risk factors [2]. In addition, all participants had been prescribed anti-osteoporotic pharmaceutical treatment. Exclusion criteria included signs of cognitive impairment, a previous diagnosis of osteoporosis, or previous treatment with anti-osteoporotic medication. The women who met the inclusion criteria were contacted by a healthcare professional, given an information letter and invited to participate in the study. Those who agreed to participate gave their name and phone number and were contacted shortly after by the researcher (CAH). One woman did not wish to continue participating when contacted prior to the second interview-round for personal reasons, and another was not reachable at the time of the last interview.

### Data source

The participants gave three interviews, for a total of 42 interviews for analysis. The first interview took place shortly after diagnosis, the second interview about six months later and the third interview approximately one year after diagnosis. These points of time were chosen because studies have shown that patients’ medical adherence stabilizes around six months after initiation [46, 47] and adjustments to live with a chronic condition may be lengthy [48]. An open approach was used to encourage the women to tell their individual stories of living with osteoporosis. An open interview guide (Additional file 1) was used [49]. Preliminary, to etch interview the women were informed of the purpose of the study and informed consent was obtained. Initially, the women were asked an open-ended question: “Please tell me about your experiences of living with osteoporosis?” The researcher would then ask clarifying questions such as “Could you please elaborate what you were telling me about xx?” to capture the individual perspective [49]. The interview guide was adjusted between interview-rounds [43]. Interviews were performed between March 2011 and August 2012 until data saturation was reached; they were tape-recorded and subsequently transcribed verbatim. Field notes were taken immediately after each interview. No medical records were available for the researchers and none of the women were patients of the authors. All information with the exception of T-scores were self-reported.

### Ethical considerations

The study was approved by the Danish Data Protection Agency (J.no. 2012-41-0875) and the National Committee on Health Research Ethics (J.no. H-C-FSP-2011_01), following the Helsinki Declaration [50]. Informed written consent was obtained at the time of enrolment and oral consent before each interview.

Individual interviews were chosen in order to explore the individual perspective in depth. The role of the first author (CAH, while she remained Ph.D. student) as an interviewer may have affected the interviews because the participants knew that she was a nurse. The participants expressed expectations of getting the opportunity to get advice during the interview, however their questions were kindly circumvented until after the interview was completed. In accordance with ethical responsibilities’ and research principles, in most cases the women were encouraged to contact their GPs or other relevant healthcare professional for clarification of the questions, as well as in cases of any other health related problem which were considered needed to be examined by a physician. On the other hand, it is also possible that the participants were more open during the interviews due to the interviewer’s healthcare profession, or due to the interview performed in a private atmosphere in their home [51]. However, the role as a nursing-researcher may have been diminished by conducting the interviews in the women’s homes, where the interaction was less affected by the interviewer’s professional status as she was a guest in the women’s homes.

Newly diagnosed patients’ are in a vulnerable situation and the experience of participation in a research study may possibly add an additional burden. When individuals have an opportunity to talk about their own experiences, they may gain meaning and understanding through telling their story which may be helpful when adapting to difficult situations [48]. This may have been the case for several of the participating women, as they spontaneously described that they had chosen to participate in the project in order to have the opportunity to gain a better understanding of osteoporosis.

Throughout the data collection the participants’ needs were looked after through attentiveness to the individual needs and wishes in relation to interview time and location.

### Analysis

**Fig. 1:**
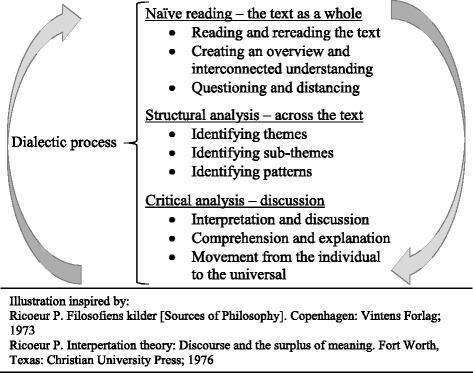
Analytic levels in the interpretation of text

According to the chosen approach, the analysis of the transcribed interviews consisted of three levels: naïve reading, a structured analysis and a critical interpretation and discussion. The analysing process took place through a dialectical movement between the parts and the whole, performed in a helical process [38–40]. In this manner, as illustrated in Fig. 1. an interpretation was performed and a new understanding of living with osteoporosis arose through the key themes and sub themes elaborated in the level of structural analysis.

The text is read several times in order to comprehend its meaning as a whole. The interpreter tries to read the text with an open mind, to allow the text to “speak”. The naïve reading is the first assumptions regarding the content of the text [39, 40]. The movement from what the text says to what it discusses was followed according to the descriptions of understanding a text in the structural analysis [39, 40] - this interpretation level is exemplified in Table 1.

**Table 1:** An example of the structural analysis and themes

Meaningful units what is said ‘Quotes’	↔	Significant units what is spoken about (primary interpretation)	↔	Sub themes	↔	Themes emissions of key themes
(1st interview round) ‘*I am confused; I do not know what might harm. (…) I want to do preventive things as much as possible. (…)Does it takes ten years to develop a fracture or how fast? How unfortunate can I be? Will something suddenly break? Many people are having pain, that is turning around in my head after I have been told that I have osteoporosis’*		being insecure when wanting to prevent uncertain consequences	Lifestyle reflection			Daily life with osteoporosis

The final interpretation is preceded by a critical analysis, continuing the dialectic process between explanation and comprehension as a discussion of relevant literature and other research findings relating to the interpretation level.

## Results

**Fig. 2:**
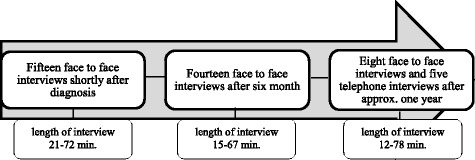
Description of the interview process

The study comprised 42 interviews with 15 women with osteoporosis ranging from 65 to 79 years (at the time of the first interview). The interviews lasted between 12 and 78 min (mean 57.48). The description of the interview process is illustrated in Fig. 2.

Most interviews were conducted at participants’ private homes, but three participants chose to give their interviews at the hospital and, at the third interview-round, five interviews were conducted by telephone. Most women had a history of hereditary osteoporosis; more than half of the women had daily back pain and all except three reported other comorbidities (primarily cancer, chronic pulmonary lung disease and collagen disease), together with some other self-reported socio-demographic information, presented in Table 2.

**Table 2:** Baseline demographics of the included 15 women

		Numbers of women
Age	65–79 years (mean 71.9)	
living with spouse		8
children	0–4 (2 women had no children)	
retired		13
employed		2
comorbidity	cancer	6
	hysterectomy	2
	collagen disease	3
	fracture	1
	cardiac disease	2
	hyper cholesterol	2
	chronic pulmonary lung disease	4
	metabolism	1
	Scheuermann’s disease	1
	more than one comorbidity	7
BMI ≤ 19		3
menopause <45y		4
hereditary disposition		10
daily pain		8
smoking/alcohol (use)	smoking: any/alcohol (use): above the recommended 14 units per week	6/1
referral to DXA scan due to	general practitioner (GP) own request to GP medical specialist	7 4 4
Exercise	follows the radio’s morning gymnastics program	5
	Line Dance	1
	rowing	1
	daily walking	9
	the municipality’s gymnastics for the elderly, led by physiotherapist	4
	always taking the stairs	3
	more than one exercise form	8

The naïve reading gave an insight about the text. Experiences of learning to live with osteoporosis were described in terms of a cognitive process of meaning creation, understanding and coming to terms with the new life circumstances and medical treatment. Descriptions were related to the development of an understanding through interpretation and reflection relating to side effects and other physical symptoms, and the interpretation and value of DXA scan results and lifestyle. This led to two key themes that emerged through the structural analysis: 1) “to become influenced by the medical treatment” and 2) “daily life with osteoporosis”. The two themes were found to describe overarching choices and strategies of living with osteoporosis. These were found to develop over time. From the descriptions shortly after diagnosis, through a further elaboration six month later and finally a pattern of living with osteoporosis approximately one year after diagnosis. An overview of the key themes and sub themes are presented in Table 3.

**Table 3:** An overview of key themes and sub themes

Key themes	Sub themes
To become influenced by the medical treatment	- taking the medication - discontinuing the medication
Daily life with osteoporosis	- interpretation of symptoms - interpretation of scanning result - lifestyle reflections

The themes will subsequently be described within the interpretation.

### To become influenced by the medical treatment

Experiences of becoming influenced by the medical treatment were described in the first round of interviews, the main focus being on practical issues of how to take the prescribed medication and how to manage side effects. This focus on side effects continued through the second and third round of interviews and a need to seek information and advice was also described. This appeared to be a cognitive process, as reflected in decisions regarding whether to take or reject the medication. The key theme was described as a trajectory illuminated by two subthemes: “taking the medication” and “discontinuing the medication”.

#### Taking the medication

At the first interview shortly after the diagnosis, taking the medication was described as a challenge: this was particularly true the first time the medication had to be taken. The action was influenced by concerns about side effects and other negative thoughts, but with a clear willingness to take medication and make it work by planning how to remember to take it once a week.

*“The first time I took it I thought it was such an execution pill, probably a really strong one. I decided to take it Friday morning before the weekend. (…) I bought a green box and I dispensed for a week. On Fridays, I have attached a big red star. (…) The star tells me I cannot eat or drink anything before or lie down and all that*” (5)

Osteoporosis is perceived as a condition that requires preventative action, as well as a disease to be taken seriously. Strategies for remembering to take the medication were described as being necessary.

Despite a continued and comprehensive focus on what were perceived as side effects and the risk of developing side effects six month after diagnosis, it appeared that developing medication-taking strategies became more natural after the conversion to life with osteoporosis took place. Six months after her diagnosis, an informant told us:

*“I take it Saturday morning when I get up. (…) I go for a walk, take a shower or walk back and forth. Then that hour has elapsed*” (12)

In some instances, the ease of preventative treatment emerged to be related to the treatment option of intravenous osteoporosis medication. One year after diagnosis, another informant claimed:

*“I get infusions. It's only once a year and it is in May. I've only got it twice so far. I feel no side effects or anything”* (13)

Despite the general ease of the treatment, thoughts about side effects were prevalent in this case. This was mainly in the third round of interviews, one year after the diagnosis, where some degree of having obtained calm and coming to terms with the medical treatment emerged. This was in regard to both the perspective of deciding to take the medication as well as in light of convincing themselves that it is the best thing to do. In contrast, few women described the ease of treatment, which consisted of an immediate decision to follow the physician’s prescription without much reflection about this issue later on.

#### Discontinuing the medication

Discontinuing the medication appeared in the text as a process based on experiences during the first year after diagnosis. Three women either stopped or did not initiate recommended osteoporosis medication; all filled their prescription at least once.

In the first interview round shortly after the diagnosis the main focus was on concerns related to side effects:

“*I did find an article.(…) There I read about making the dentist aware of it (…) It has something to do with the healing process of the jaw (…) If they [the tablets] are uncomfortable in some ways’ I am not sure (pause). I believe the medicine is a little harsh”* (3)

These concerns were described in connection to new knowledge obtained through seeking information about the medication. Moreover, some signs of insecurity related to the decision could be traced in this stage of the process. Six months later, concerns about the medication have developed in terms of own experiences of discomfort or side effects leading to reflections on the decision to discontinue the medication:

*“I felt weird in my stomach (…) I did not sleep well the first nights. (…) Maybe it's mental, but I really don't like taking them. I got the feeling that they were unpleasantly rough to my stomach. (…) Then I stopped taking them. I was wondering if they are so harsh, is it then worth taking them?”* (15)

Not being worried about treatment side effects can be a relief. On the other hand, some insecurity regarding the decision to discontinue the medication is interpreted in the text. The interpretation of a degree of insecurity regarding the decision is strengthened by narratives of continuing to seek information and advice from various sources at this stage. One year after diagnosis at the third round of interviews, a certain calm related to coming to terms with the decision to discontinue the medication was found.

“*I’ve got heart problems (…) it has overlapped with it [osteoporosis]. It is more important that the heart beats than I might have to sit in a wheelchair. I have chosen not to take the medication [anti-osteoporotic]. I am instead trying to do everything else to strengthen my bones”* (3)

In this particular case, the experience of the decision was influenced by a larger life event of dealing with a potential heart failure.

Another perspective of the decision process regarding the medical treatment was also found in the text, as one woman described persistent resistance towards taking the medication in the first interview. Throughout the second interview, she maintained a strong resistance and a firm decision not to take the medication, although there was the twist of doing as she “was told”.

*“I'm not bothered by anything. I do not take those stupid pills. No I don't. I hate to take pills. The GP who gave them to me said that I might get side effects. I said ‘you know what? I do not want them it works just fine’. (…) On the other hand I am very (pause) I obey the doctor [she bought them anyway]”* (11)

A pronounced reserved attitude towards taking medication was a general finding, although this example was the most distinct.

### Daily life with osteoporosis

The theme “daily life with osteoporosis” was supported in the text by descriptions of a trajectory and evolution of experiences related to three subthemes: “interpretation of symptoms”; “interpretation of scanning result” and “lifestyle reflections” (Table 3).

#### Interpretation of symptoms

The sub theme “interpretation of symptoms” contains experiences relating to the interpretation of osteoporosis through finding explanations and developing understanding of the evolution of increased or reduced symptoms of side effects, back pain and other physical symptoms. The individual interpretation and understanding of osteoporosis as being the reason for what had been causing back pain for many years began shortly after the diagnosis:

*“It's [osteoporosis] apparently something that comes sneaking (…) I have not been able to understand why I got sore back again and again. I HAVE had back pain as if it explodes”* (1)

The diagnosis is found to generate a cognitive process which brings a new perspective when learning to live with osteoporosis. In addition, trust that the physician would be able to provide help in understanding and interpreting the symptoms also appeared:

*“My GP has promised to help me find out about the pain I have. Whether it is associated with osteoporosis or whether it is something else. (…) He has to tell me what to do. (…) I have experienced that one can learn to live with pain. I am not just sitting in a chair”* (10)

Learning to live with osteoporosis is found to be based on an acceptance of the diagnosis generated though an understanding of symptoms as being related to osteoporosis. This process began shortly after the diagnosis but was generally clarified in the third round of interviews by descriptions of feelings verifying the diagnosis. When the symptoms declined, it was interpreted as being due to the pharmaceutical treatment stressing that the diagnosis was real. Approximately one year after the diagnosis, an informant stated:

*“I've been so ill, so I couldn't even pull the quilt up. Finally, I got it, ‘forsteo’. I have taken it for a year now. (…) The past one and a half months I sometimes wake up in the morning without having backache*” (9)

This kind of reflection on symptoms emerged to be a common part of the cognitive process of accepting a future life with osteoporosis.

#### Interpretation of the DXA result

The sub theme *interpretation of the scan result* contains descriptions of the interpreted value along with the understanding of the scan result. In several cases, it appeared to be difficult for the women to understand the result of their scan. In the first interview, one woman said:

*“Approximately three years ago I decided to get it [osteoporosis] examined, I was over the line, but far down [on the scale]. (…) In this fall I got it reassessed. (…) the numbers [from the DXA scan result] were almost the same but finally it was addressed (…) they said I have osteoporosis”* (14)

Despite the participants’ difficulties in interpreting results from DXA scans, the scan results were perceived as being of significance for the development of understanding and acceptance of the diagnosis. It appeared in the text that these results were commonly used in the process of learning to live with osteoporosis. Six months after the diagnosis one woman noted:

“*My sister was here recently. We compared her pictures with mine, and the values. (…) I don’t know when I am having the next scan. (…) I don’t know how fast you can see an effect [of the treatment on a DXA scan result]”* (4)

The scanning result served as a form of verification of the diagnosis, and was expected to confirm the effects of the treatment. About one year after the diagnosis, an informant claimed:

*“In December I’ll have to sign up for a new scan. Then we'll see if it shows any change. Some improvement hopefully (pause) or maybe there has been very little change”* (2)

In addition, expectations regarding the next scanning result were found to be of value, since this was talked about as being the “proof” of whether the treatment was effective. As clarified by the quote, throughout the text future expectations were related to the hope of visual signs of improvement.

#### Lifestyle reflections

The sub theme “lifestyle reflections” was found to be a process mediating the acceptance and adaption to learning to live with osteoporosis. This was described in the text as experiences of daily life shortly after the diagnosis (at the first rounds of interviews), focusing on information or thoughts related to lifestyle advice when living with osteoporosis. This was further elaborated through the second round of interviews which primarily focused on changes related to diet and physical activity. In the third round of interviews, we found continued reflections relating to diet and physical activity as well as prior lifestyle. The process is illuminated by descriptions of the potential insecurity of a future life with osteoporotic consequences and how to prevent these consequences. Shortly after the diagnosis:

“*I am confused; I do not know what might harm. (…) I want to do preventive things as much as possible. (…)Does it takes ten years to develop a fracture or how fast? How unfortunate can I be? Will something suddenly break? Many people are having pain. That is turning around in my head after I have been told that I have osteoporosis”* (8)

The fear of possible future consequences of osteoporosis emerged throughout the text and was found to both mediate and challenge the process of acceptance. Reflections and concerns about “doing the right thing” were often expressed in conjunction with experiences of differing and confusing information and perceptions of lifestyle advice (in this example intake of supplemented intake of calcium). At the second interview, an informant claimed:

*“I have always been exercising, I have been eating healthily and I do not smoke and I do not drink. I think I have done what I could to have a healthy life. (…) I am always thinking whether I get enough calcium. At the pharmacy they say I have to take three tablets a day, otherwise I have been told that I have to take two a day, what am I supposed to do? (…) I am sort of pushing it in front of me all the time. Am I doing it right? Am I generating a hunched back or get a fracture? What will happen?”* (15)

Coming to terms with new life circumstances and trying to do ‘the right thing’ was made more difficult by what was perceived as confusing and differing information. Moreover, understanding and acceptance of the diagnosis was obtained by interpretation and explanations through narratives of a former lifestyle as exemplified in the third round of interviews:

*“I've known for many years that I probably had it [osteoporosis]. As a child I did not like milk. I had some allergies. (…) I still do not like milk therefore it is important to incorporate calcium in my food other than calcium tablets. I eat ten to twelve almonds every morning. (…) I eat sardines in oil and tuna in oil they are a little fat and have vitamin D”* (7)

Part of the process of coming to terms to living with osteoporosis involved seeking an explanation to why the patients had developed osteoporosis. Most cases included reflections on “why”.

Exceptions from the cognitive process of learning to live with osteoporosis were found in a small number of women who appeared to be occupied with other issues, such as having a severe comorbidity, taking care of an ill spouse or just perceiving it as a “natural thing”:

*“We've a good life. He [the husband] is not able to talk. He has aphasia and a half-side paralysis. His mood is good. We have learned to live with that side of our lives, and that is more important than osteoporosis. (…) I do not know what to do about it, so I don't do anything. I missed instructions. I hardly know what osteoporosis is. That's probably why it does not make as much impression on me”* (6)

The process of learning to live with osteoporosis was generally found to be highly influenced by the perception of pharmaceutical treatment. The understanding and interpretation of information and medical advice, together with the interpretation of symptoms and scan results were, together with the interviewees’ prior lifestyle, exposed to a deeper reflection. The perceptions of a future DXA scan emerged as an important life event, with expectations and hope for an improved result.

## Discussion

The study adds knowledge to how women, who had not experienced an osteoporosis fracture at the time of diagnosis, learn to live with osteoporosis after being prescribed pharmaceutical treatment. The experiences described a multifaceted process that is highly influenced by the medical treatment and daily life. The interviewees’ daily lives were found to be adapted according to understanding and interpreting of a bigger picture of lifestyle, including bodily “signals” such as past and current symptoms, pain, side effects (or what were perceived as being side effects) as well as the scan results. Moreover, accepting and understanding of the diagnosis was found to be dependent on the adaptation of daily life, and vice versa.

Medical aspects such as having to take medication and side effects have an important role in life with osteoporosis and perhaps especially before a known osteoporotic fracture occurs, since the medication is tangible and has to be managed in one way or another. It may be assumed that the reason why most women are continuously focused on side effects during the first year after diagnosis is due to their understanding of benefits of having been diagnosed and understanding of the importance of the pharmaceutical fracture preventative therapy in decreasing the risk of osteoporotic fractures. Our findings on this central issue contribute to the findings of other studies dealing with treatment-requiring osteoporosis, the initiation of pharmaceutical treatment as well as persisting and complying with anti-osteoporotic therapy [8, 21, 29, 44, 52–54]. Yu et al. specifically investigated reasons for not initiating osteoporosis therapy [54]. They found that 37.6% of 117 survey responders did not initiate recommended osteoporosis medication due to concerns over side effects in 77.3% of the cases. These findings may be seen as consistent with findings in the current study.

The current findings of accepting and understanding the diagnosis are in accordance with studies by Kralik et al. [55, 56] who explored women’s narratives of “being” diagnosed with a long-term illness [55] and the process of “moving on” when living with a chronic illness or condition [56]. The studies reported that many women experienced receiving the medical diagnosis of their long-term illness as a memorable event in their lives; the experience of getting a diagnostic label was comprehensive and should not be underestimated [55]. Additionally, it was reported that “moving on” with a chronic illness or a condition is a complex process of learning, finding meaning and the redefining of self - a unique journey for each person depending upon their particular situation and context [56]. This was in line with what the women in our study described when reflecting on their lifestyle and trying to understand why they have developed osteoporosis, and how to live their daily lives with this chronic disease. This process may not be specific to osteoporosis, and healthcare professionals treating osteoporosis patients may benefit and learn from studies on other chronic diseases

Similarities may be drawn to the grounded theory study of “healthy risk awareness” [27], which develops through an emotional cognitive process of acceptance. Being motivated to act in a preventative way was found to impact upon acceptance, which underwent a development through the fear of having fragile bones [27]. Descriptions of fear in the current study were primarily elaborated through concerns related to the medical treatment, side effects and making the right choice, but also concerns regarding possible fractures [44]. These worries were addressed by developing strategies and making adjustments in everyday life. This may be understood as being generated by fear being used to develop “healthy risk awareness” as found in the study by Hjalmarson et al. Developing strategies by taking control has been found to be a way of addressing a negative outcome such as having fractures [8]. Moreover, it was found that strategies have been developed when patients accepted their need for treatment [28]. In the current study, acceptance appeared to be mediated by the perception of the medical treatment. The women were confused by differing information and medical advice from across various sources. They sought to understand the information and interpret their symptoms, side effects and the results of their scan within a bigger picture of daily life with osteoporosis. Significant parts of this understanding and interpretation of the accepting process were related to the decisions regarding taking or discontinuing the medication. The individual perception appears to be an important factor, which should be highlighted when dealing with osteoporotic fracture prevention. One of the most pronounced challenges is to uncover factors which may increase adherence to anti-osteoporotic treatment. In a large national register-based study, it was found that 38.7% of the patients stopped treatment within the first year [23]. Although this is of a smaller scale when compared to international findings [21, 22, 24, 25], it is still a problem from a fracture prevention perspective [16]. The decision-making process has been reported in other studies to be an emotional and cognitive learning process [27]; a sense-making process [8]; and a process of trial and error to find useful strategies [28]. One common limitation of these and other qualitative studies that investigate the decision-making process regarding medication use of individuals with osteoporosis appears to be the lack of studies with longitudinal design following patients’ experiences over time (no qualitative studies on this matter were found). The multifaceted factors which were found to be related to decision-making in the current study may be the explanation of why it was found to be a matter of an immediate decision for some, while for others, it was a longer process that developed over time. This finding may be supported by the study of challenges regarding transition to a life with chronic illness [56].

### Implications for research and practice

Interventions targeting osteoporotic fracture prevention that encourage collaboration between patients and healthcare professionals may incorporate approaches of shared decision-making or other equivalent approaches for persistence of use enhancement and patient education. Shared decision-making has been found to be the most effective therapeutic option to increase compliance and persistence to medical treatment, together with fostering greater patient satisfaction and improving the healthcare processes and outcomes for patients [57]. It may, however, be difficult to implement this model, as healthcare professionals may find it hard to take a health-(and an individual) oriented stance and focus on the particular patient’s reaction to the illness in daily life, as it is different from the disease-oriented perspective [58]. Despite potential difficulties, the current study highlights the importance of making room for patients’ individual narratives to strengthen their empowerment. The stories told by patients may naturally find their place as a part of the nursing consultation in the outpatient clinic. A participant-oriented direction requires innovation among healthcare professionals.

Further research is needed. We therefore suggest that further intervention studies comparing current practice to more individualized plans for decision-making adapting to individuals’ need for access to professional advice (such as nursing consultations, telephone hotline or telemedicine [57–59]) may be beneficial when targeting osteoporotic fracture prevention and learning to live with osteoporosis.

Other perspectives, based on the current study results, may provide the opportunity of developing targeted register-based studies. The current study suggests that there is a need for more targeted surveys of patients and healthcare professionals examining their attitude and assessments of information of the treatment, the condition, socio-demographic and health-related factors etc.

### Strengths and limitations of the study

The reporting of this qualitative study has sought to meet the recommendations of The Consolidated criteria for reporting qualitative research (COREQ): a 32-item checklist for interviews and focus groups (Additional file 2) [60]. This instrument is recommended by BioMed Central. The study comprised of 42 interviews involving 15 participants. This number may be seen as a strength of the study. Additionally, the longitudinal design is considered to provide a thorough exploration of the research question [43]. Since the survey only included women over the age of 65 who voluntarily participated in the study, it is possible to assume, for instance, that younger woman or those who chose not to participate would have brought a different perspective to the results.

Five interviews were conducted by telephone - this was chosen at the convenience of the participants to ensure participation. Telephone interviews can be an effective method of data collection when the interviewer is aware of the challenges involved [61]. When conducting telephone interviews the ability to observe non-verbal communication is missing; instead, however, the tone of the voice can be taken into consideration to conjure a picture of the individual [62]. Based on the previous interactions, we did not get the impression that the interviews by telephone affected the data.

The interviews in this study allowed for open, nuanced descriptions of various aspects of women’s lives after the diagnosis when medical treatment was prescribed. The appearance of the phenomenon “life with osteoporosis” was enabled.

No qualitative study is without the “touch” or influence of the investigation itself or the researcher as an “interacting” individual. When conducting qualitative research, it is crucial to reflect upon possibilities of how the researcher affects the process or whether such an effect can be prevented, during data collection, analysis and interpretation [63]. During interviews, the first author sought to be aware of own preconceptions, not posing leading questions but instead giving the women the opportunity to tell their story and time to pause during its narration, after which the women were encouraged to elaborate upon something they had mentioned with the purpose of clarifying the meaning attached to the individual experience.

Analysis and interpretation were discussed with fellow researchers (CAH: female, AB: male, HK: female and BDP: female) during all three levels of the interpretation to enhance trustworthiness. Involvement of multiple researchers is recommended when conducting qualitative research, as this might strengthen the design of a study. During the analysis process and interpretation multiple researchers may supplement and contest each other’s statements which may enrich and qualify the analysis [49, 63].

## Conclusion

Women’s experiences of living with osteoporosis the first year after diagnosis were characterized by a complex process of learning to live with their new life circumstances. This process was found to be highly influenced by finding strategies that encompass taking the medication, side effects or concerns about side effects, the acceptance and interpretation of scan results, symptoms and the diagnosis, as well as decision-making. There is a need for communication tailored to individual needs, since the uncertainty of living with the new life circumstances may be overwhelming and may lead to inappropriate decisions which could affect pharmaceutical treatment. Improved individualized support for women in the process of learning to live with osteoporosis may be improved through using a health-oriented stance. More research is needed from the perspective of both health promotion and prevention.

## Additional files

Additional file 1. This file contains the applied open interview guide. (DOC 36 kb)
Additional file 2. This file contains the checklist “The Consolidated criteria for reporting qualitative research (COREQ): a 32-item checklist for interviews and focus groups”. This instrument is recommended by BioMed Central. (DOCX 20 kb)

## Abbreviations

BMD: Bone mineral density
DXA: Dual-energy X-ray absorptiometry
