# Supplementary material for: Women’s lived experiences of learning to live with osteoporosis: a longitudinal qualitative study
Source: BMC Womens Health. 2017 Mar 9;17:17. doi: 10.1186/s12905-017-0377-z (PMC5345268; doi:10.1186/s12905-017-0377-z)
Supplement: Additional file 1. — This file contains the applied open interview guide. (DOC 36 kb) [file 12905_2017_377_MOESM1_ESM.doc]

| **AREA** | **QUESTIONS**  **First interview round** | **QUESTIONS**  **Second and third interview round** |
| --- | --- | --- |
| Initial: The interview will be focused on how you experience living with osteoporosis and what you perceive as important in your everyday life | Would you please start by telling a little about yourself?  • Age, marital status, children, education, where you live and so on? |  |
| Newly diagnosed with osteoporosis | How long has it been since you were told that you have osteoporosis?  • How did you get the message? |  |
| Life with osteoporosis | **How do you experience life with osteoporosis?**   - What have you learned so far? | **How do you experience life with osteoporosis?**   - What have you learned so far? |
|  | How do you experience the fact that you have to live with osteoporosis?  • Is your life like before you got the diagnosed? | Have there been some significant changes since we last talked in relation to your life with osteoporosis?  • Do you live your life differently now than you did six months ago? |
|  | What are your thoughts about osteoporosis?  • Have you had to make some choices? | What are your thoughts about osteoporosis? |
|  | Is there something that has been particularly significant or meaningful related to your newly diagnosed osteoporosis?  • Please tell about the trajectory? | Is there anything that has had particular importance to you and the way you choose to live your life? |
| Final | Is there anything you would like to elaborate on or tell about before we finish the interview? | Is there anything you would like to elaborate on or tell about before we finish the interview? |
