# Supplementary material for: Women’s lived experiences of learning to live with osteoporosis: a longitudinal qualitative study
Source: BMC Womens Health. 2017 Mar 9;17:17. doi: 10.1186/s12905-017-0377-z (PMC5345268; doi:10.1186/s12905-017-0377-z)
Supplement: Additional file 2. — This file contains the checklist “The Consolidated criteria for reporting qualitative research (COREQ): a 32-item checklist for interviews and focus groups”. This instrument is recommended by BioMed Central. (DOCX 20 kb) [file 12905_2017_377_MOESM2_ESM.docx]

| Domain 1: Research team and reflexivity  Personal Characteristics |  |
| --- | --- |
| 1. Interviewer/facilitator Which author/s conducted the interview or focus group? | **Yes**  **Line 201: The role of the first author (CAH, while she remained Ph.D. student) as an interviewer**  **Line 611: During interviews, the first author sought to be aware of** |
| 2. Credentials What were the researcher’s credentials? E.g. PhD, MD | **Line 4 - 6** |
| 3. Occupation What was their occupation at the time of the study? | **Line 4 - 6** |
| 4. Gender Was the researcher male or female? | **Line 617: with fellow researchers (CAH: female, AB: male, HK: female and BDP: female)** |
| 5. Experience and training What experience or training did the researcher have?  Relationship with participants | **Line 4 – 6**  **Line 191: No medical records were available for the researchers and none of the women were patients of the authors** |
| 6. Relationship established Was a relationship established prior to study commencement? | **No.**  **Line 171: Those who agreed to participate gave their name and phone number and were contacted shortly after by the researcher (CAH).** |
| 7. Participant knowledge of the interviewer What did the participants know about the researcher? e.g. personal goals, reasons for doing the research | **Line 203: the participants knew that she was a nurse.**  **Line 170: …given an information letter and invited**  **Line 184: Preliminary, to etch interview the women were informed of the purpose of the study and informed consent was obtained.** |
| 8. Interviewer characteristics What characteristics were reported about the interviewer/facilitator? e.g. Bias, assumptions, reasons and interests in the research topic | **Line 202: The role of the first author (CAH, while she remained Ph.D. student) as an interviewer may have affected the interviews because the participants knew that she was a nurse.**  **Line 213: the role as a nursing-researcher may have been diminished by conducting the interviews in the women’s homes, where the interaction was less affected by the interviewer’s professional status as she was a guest in the women’s homes** |
| Domain 2: study design  Theoretical framework |  |
| 9. Methodological orientation and Theory What methodological orientation was stated to underpin the study? e.g. grounded theory, discourse analysis, ethnography, phenomenology, content analysis  Participant selection | **Line 143: The French philosopher Paul Ricoeur`s work is commonly regarded as a bridge between the philosophies of phenomenology and hermeneutic**  **Line 229: the analysis of the transcribed interviews consisted of three levels: naïve reading, a structured analysis and a critical interpretation and discussion. The analysing process took place through a dialectical movement between the parts and the whole, performed in a helical process** |
| 10. Sampling How were participants selected? e.g. purposive, convenience, consecutive, snowball | **Line 161: Fifteen participants were included consecutively according to inclusion and exclusion criteria** |
| 11. Method of approach How were participants approached? e.g. face-to-face, telephone, mail, email | **Line 160: Women were contacted when they attended a DXA scan**  **Line 252: Most interviews were conducted at participants’ private homes, but three participants chose to give their interviews at the hospital and, at the third interview-round, five interviews were conducted by telephone.** |
| 12. Sample size How many participants were in the study? | **Line 161: Fifteen participants were included** |
| 13. Non-participation How many people refused to participate or dropped out? Reasons?Setting | **Line 172: One woman did not wish to continue participating when contacted prior to the second interview-round for personal reasons, and another was not reachable at the time of the last interview** |
| 14. Setting of data collection Where was the data collected? e.g. home, clinic, workplace | **Line 252: Most interviews were conducted at participants’ private homes, but three participants chose to give their interviews at the hospital and, at the third interview-round, five interviews were conducted by telephone.** |
| 15. Presence of non-participants Was anyone else present besides the participants and researchers? | **No.**  **Line 212: the interview performed in a private atmosphere in their home** |
| 16. Description of sample What are the important characteristics of the sample? e.g. demographic Data collection | **Table 2** |
| 17. Interview guide Were questions, prompts, guides provided by the authors? Was it pilot tested? | **Line 189: The interview guide was adjusted between interview-rounds** |
| 18. Repeat interviews Were repeat interviews carried out? If yes, how many? | **Yes.**  **Line 177: The participants gave three interviews** |
| 19. Audio/visual recording Did the research use audio or visual recording to collect the data? | **Line 191: tape-recorded and subsequently transcribed verbatim.** |
| 20. Field notes Were field notes made during and/or after the interview or focus group? | **Line 192: Field notes were taken immediately after each interview.** |
| 21. Duration What was the duration of the interviews or focus group? | **Line 177: The first interview took place shortly after diagnosis, the second interview about six months later and the third interview approximately one year after diagnosis.** |
| 22. Data saturation Was data saturation discussed? | **Line 191: until data saturation was reached**  **Line 596: younger woman or those who chose not to participate would have brought a different perspective to the results** |
| 23. Transcripts returned Were transcripts returned to participants for comment and/or correction? | **No** |
| Domain 3: analysis and findingsz  Data analysis |  |
| 24. Number of data coders How many data coders coded the data? | **No coding were not a part of the chosen analyzing method**  **Line 229: the analysis of the transcribed interviews consisted of three levels: naïve reading, a structured analysis and a critical interpretation and discussion. The analysing process took place through a dialectical movement between the parts and the whole, performed in a helical process** |
| 25. Description of the coding tree Did authors provide a description of the coding tree? | **Not relevant** |
| 26. Derivation of themes Were themes identified in advance or derived from the data? | **Derived from Data during**  **Line 234: an interpretation was performed and a new understanding of living with osteoporosis arose through the key themes and sub themes elaborated in the level of structural analysis.** |
| 27. Software What software, if applicable, was used to manage the data? | **Not relevant** |
| 28. Participant checking Did participants provide feedback on the findings? Reporting | **No** |
| 29. Quotations presented Were participant quotations presented to illustrate the themes / findings? Was each quotation identified? e.g. participant number | **Yes identified by participant number** |
| 30. Data and findings consistent Was there consistency between the data presented and the findings? | **yes** |
| 31. Clarity of major themes Were major themes clearly presented in the findings? | **yes** |
| 32. Clarity of minor themes Is there a description of diverse cases or discussion of minor themes? | **yes** |
